# Impact of GFR on Mortality Risk After Biliopancreatic Diversion: Challenges and Pitfalls for the Clinician

**DOI:** 10.1007/s11695-025-08239-z

**Published:** 2025-09-18

**Authors:** Elisa Russo, Elvina Lecini, Antonio Bottino, Valerio Abeti, Lucia Maccò, Pasquale Esposito, Gian Franco Adami, Francesco Saverio Papadia, Francesca Viazzi

**Affiliations:** 1 University of Genoa, Genoa, Italy; 2 IRCCS Ospedale Policlinico San Martino, Genoa, Italy

**Keywords:** Glomerular filtration rate, Obesity, Bariatric surgery, Biliopancreatic diversion, Malnutrition, Mortality

## Abstract

**Background:**

Assessing kidney function in bariatric patients remains challenging, as both obesity and malnutrition act as confounding factors. Although short-term cardiovascular and renal benefits of biliopancreatic diversion (BPD) have been documented, the long-term effects of estimated glomerular filtration rate (eGFR) improvement remain unclear.

**Methods:**

To compare different eGFR formulas based on creatinine and to evaluate the relationship between short- and long-term eGFR changes and mortality risk in patients undergoing BPD.

**Results:**

284 patients were enrolled. Mean pre-surgery BMI, creatinine, and eGFR were 47.0 ± 9.3, 0.87 ± 0.21 mg/dl, and 75.3 ± 15.9 mL/min/1.73m^2^, respectively. During a mean follow-up of 16 ± 9 years, 40 patients (16%) died. BMI decreased by -3.7 ± 5%/year, while unadjusted eGFR increased by + 3.1 ± 7.8 mL/min/m^2^/year. Significant differences in GFR estimates were observed, especially at baseline. In an adjusted Cox model, greater weight loss was associated with increased mortality, independent of baseline BMI (HR 2.48 [95%CI 1.01–6.07], *p* = 0.047). An increase in eGFR during the first year following surgery was associated with a reduced risk of mortality (HR 0.96 [95% CI 0.93–0.98], *p* = 0.002), suggesting that a lack of short-term improvement in eGFR should be a red flag for clinicians. Conversely, a sustained increase in eGFR beyond the first year was associated with a higher risk of mortality (HR 1.15 [95% CI 1.15–1.48], *p* < 0.001).

**Conclusions:**

Estimating GFR in bariatric surgery remains challenging. The findings illustrate a time-dependent impact of eGFR improvement on mortality risk following biliopancreatic diversion, highlighting the importance of personalized postoperative monitoring and nutritional management.

**Supplementary Information:**

The online version contains supplementary material available at 10.1007/s11695-025-08239-z.

## Introduction

Obesity is a major global health issue, significantly contributing to increased cardiovascular (CV) [1] and overall mortality risk [2]. The prevalence of obesity has reached epidemic proportions, leading to a rise in type 2 diabetes (T2D), hypertension, and chronic kidney disease (CKD), which together elevate the risk of both CV disease and mortality [3]. The impact on kidney function is particularly concerning, as obesity accelerates CKD progression and can lead to end-stage renal disease (ESRD), further compounding health risks [4]. In a global database including over 5.4 million healthy individuals, the risk of decline in GFR progressively and independently increased with rising of the BMI [5].

Bariatric surgery has emerged as an effective intervention for severe obesity, offering substantial and sustained weight loss, which translates into a wide range of health benefits [6, 7]. In addition to improving metabolic conditions, bariatric surgery has shown positive effects on kidney health, reducing proteinuria, and potentially slowing CKD progression and even reversing early renal damage [7, 8]. Procedures such as biliopancreatic diversion (BPD) and gastric bypass not only improve body mass index (BMI) and reduce obesity-related complications but also address hyperfiltration and other renal stressors associated with obesity. By reducing weight and improving metabolic profiles, bariatric surgery lowers overall and cardiovascular mortality risks, enhancing quality of life and long-term outcomes. At the same time, patients undergoing bariatric surgery may develop a risk of malnutrition, particularly those with CKD [9, 10]. In bariatric surgery, an accurate assessment of kidney health plays a crucial role in evaluating surgical risk, guiding postoperative recovery and determining long-term renal outcomes [11]. Accurately assessing GFR in patients with severe obesity, especially in the context of bariatric surgery, presents several challenges [12]. First, widely used equations for estimating GFR, like the CKD Epidemiology Collaboration (CKD-EPI) and the Modification of Diet in Renal Disease (MDRD) equations, were developed based on older datasets from populations in which severe obesity was far less prevalent [13]. Additionally, large weight loss itself influences creatinine levels, regardless of actual kidney function, complicating the accuracy of these measurements [14].

A key issue in assessing GFR after bariatric surgery is the indexing of GFR to body surface area (BSA), as BSA changes significantly with major weight loss. Typically, both measured and estimated GFR (eGFR) are adjusted to a standard BSA of 1.73 m^2^, based on the assumption that GFR is linked to kidney mass, which correlates with body mass in mammals [15]. This adjustment reduces variability in kidney function measurements among healthy people. However, in individuals with severe obesity, BSA indexing results in notably lower GFR values, raising questions about its appropriateness for post-bariatric surgery patients [16, 17].

In this study, the primary objective was to examine the performance of indexed and non-indexed eGFR using creatinine levels, comparing the different available formulas. The second aim was to assess the impact of GFR changes during the follow-up study period on the outcomes of patients who underwent biliopancreatic diversion.

## Materials and Methods

### Study Population

A total of 284 adults with BMI ≥ 35 kg/m^2^ undergoing biliopancreatic diversion (BPD) at San Martino Hospital of Genoa from 12th May 1976 to 9th April 1994. 254 patients had complete information on clinical and biochemical data for at least 2 years from surgery.

The primary outcome was all-cause mortality.

### Estimating of GFR

Previously published Chronic Kidney Disease Epidemiology (CKD-EPI) Collaboration formula [13], Cockcroft–Gault (CG) [18], Salazar-Corcoran [19] and European Kidney Function Consortium (EKFC) [20], estimating equations were used to estimate GFR using creatinine levels. CG equation was estimated using both LBW (CG-LBW equation) and real weight. CKD-EPI formula, conventionally standardized to a BSA of 1.73 m^2^ [21], was also unadjusted for standard BSA and indexed for real BSA.

The main results are shown for the CKD-EPI formula non-indexed, which has been validated and seems to be the most appropriate equation, from a methodological point of view [22]. Change in GFR (CKD-EPI non-indexed) at the 1-year time point (ΔGFR_1y_), and the yearly change in GFR over time (ΔyGFR) were calculated.

### Statistical Analysis

Normally distributed variables are presented as mean ± standard deviation (SD) and compared using an independent or paired *t-test* as appropriate. Comparisons of proportions were made using the x^2^-test or Fisher’s exact test whenever appropriate.

Kaplan–Meier and log-rank test methods were used to estimate and compare survival curves. Cox proportional hazard regression was used to estimate the hazard ratio and 95% confidence intervals (CIs) for the relationship between yearly GFR change and several clinical and laboratoristic parameters to primary end point. Covariates that were considered, by means of clinical criteria, potential confounders of the relationship between change GFR and mortality (such as malnutrition, BMI at baseline and during follow-up, age and gender) were included in the multivariate models. Statistical analyses were performed using Stata (version 14.2, StataCorp LP; College Station, TX, USA). P-value less than 0.05 was considered statistically significant.

## Results

Our cohort had a mean follow-up of 16.2 ± 9.1 years. The overall mean age was 36.1 ± 10.9 years, with 73.5% of the patients being women. Regarding comorbidities, 60% had a diagnosis of hypertension, and 43% had diabetes. The mean pre-surgery BMI, creatinine, and eGFR (estimated by unindexed CKD-EPI formula) were 47.0 ± 9.3, 0.87 ± 0.21 mg/dL, and 75.3 ± 15.9 mL/min/m^2^, respectively.

No substantial differences were observed in the clinical and laboratory characteristics of the study population divided into baseline GFR tertiles (Table 1). Along with the growth in tertiles, patients were shown to be younger and to have a less severe grade of obesity. Hypertension was more prevalent in the first tertile, whereas diabetes was more frequent in the second tertile. A higher BMI (49.5 ± 10.0) was associated with a lower average eGFR (*p* = 0.0077).

**Table 1:** The main clinical characteristics of whole cohort and according to baseline eGFR tertiles

	All	Baseline eGFR 1° Tertile	Baseline eGFR 2° Tertile	Baseline eGFR 3° Tertile	p
	(N = 254)	< 89 (ml/min/m^2^)	90–107 (ml/min/m^2^)	> 107 (ml/min/m^2^)	
General clinical characteristics					
Age [years]	36.1 ± 10.9	41.2 ± 11.2	35.9 ± 9.5	31.2 ± 9.5	< 0.001
Gender, female	73.5	78.8	61.9	78.6	0.02
Weight pre [kg]	127 ± 28	135 ± 30	127 ± 26	118 ± 23	< 0.001
BMI pre-surgery [Kg/m^2^]	47.0 ± 9.3	49.5 ± 10.0	46.4 ± 8.2	45.2 ± 9.0	0.01
BSA pre-surgery [m^2^]	2.26 ± 0.26	2.33 ± 0.26	2.27 ± 0.27	2.17 ± 0.21	< 0.001
Comorbidities					
Hypertension [%]	60.1	67.1	58.3	54.8	0.24
SBP [mmHg]	146 ± 27	152 ± 30	146 ± 25	140 ± 23	0.02
Diabetes, [%]	43.0	43.5	47.6	35.7	0.28
Laboratory tests at baseline					
Glycemia [mg/dL]	104 ± 37	108 ± 40	109 ± 44	96 ± 23	0.03
Total cholesterol [mg/dL]	211 ± 45	208 ± 41	215 ± 48	210 ± 45	0.60
HDL- cholesterol [mg/dL]	44 ± 17	43 ± 14	46 ± 21	42 ± 13	0.47
LDL-cholesterol [mg/dL]	139 ± 45	138 ± 39	133 ± 44	147 ± 51	0.43
Triglycerides [mg/dL]	148 ± 81	145 ± 64	164 ± 109	135 ± 61	0.07
Hemoglobin [mg/dL]	14.5 ± 7.8	14.4 ± 7.7	15.4 ± 11.1	13.6 ± 7.8	0.35
Platelets [× 10^9^/L]	263 ± 71	253 ± 77	256 ± 62	281 ± 70	0.02
Immunoglobulins [g/L]	1.27 ± 0.56	1.35 ± 0.82	1.19 ± 0.41	1.28 ± 0.27	0.24
Serum Albumin [g/dL]	4.1 ± 0.5	4.1 ± 0.6	4.1 ± 0.4	4.1 ± 0.5	0.62
Proteins [g/dL]	7.22 ± 0.60	7.23 ± 0.55	7.21 ± 0.62	7.21 ± 0.61	0.97
Creatinine [g/dL]	0.87 ± 0.21	1.02 ± 0.24	0.88 ± 0.13	0.71 ± 0.12	< 0.001
GFR (estimated by unindexed CKD-EPI) [ml/min/m^2^]	75.3 ± 15.9	58.5 ± 8.6	75.0 ± 3.1	92.6 ± 9.8	< 0.001

Abbrevations: eGFR, estimated glomerular filtration rate; BMI, body mass index; BSA, body surface area; SBP, sistolic blood pressure

**Fig. 1:**
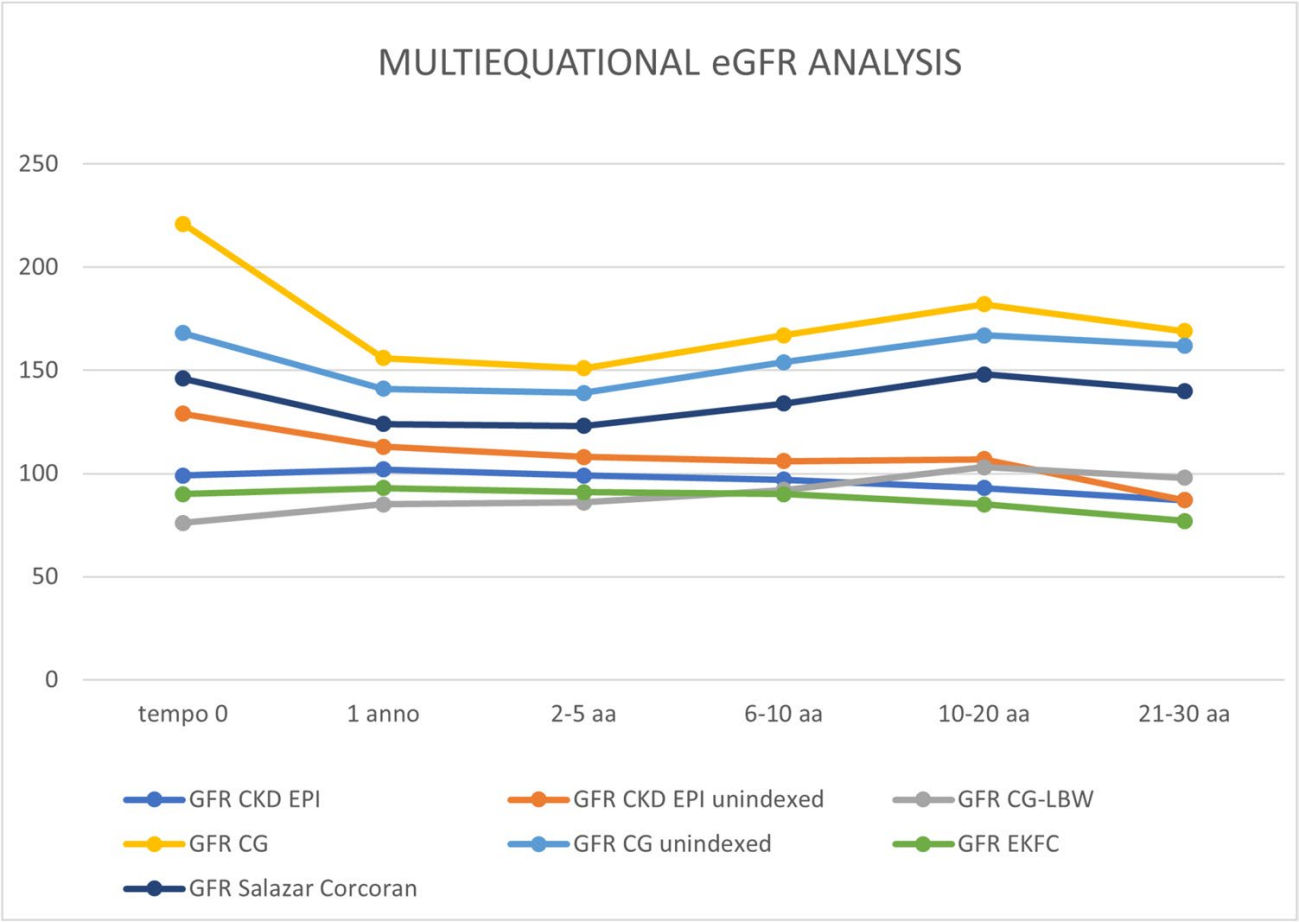
Multiequational eGFR trend during follow up. Abbreviations: BSA, body surface area; EKFC: European Kidney Function Consortium; eGFR: estimated glomerular filtration rate; CKD EPI: Chronic Kidney Disease-Epidemiology Collaboration equation; LBW, lean body weight

We analyzed estimated GFR using different equations available over the three decades of follow-up. We identified five follow-up points: the first year, 3rd, 7th, 15th, and 25th years (Fig. 1). Supplemental Table 1 specifies the formulas used and the methods for unindexing for standard BSA and indexing for real BSA (Supplemental Table 1).

Patients with a ΔeGFR greater than 20% after the first year were those with lower eGFR at baseline who showed a mean higher eGFR one year after the intervention and who benefited from a greater reduction in BMI in the first year and as annual ΔBMI (*p* < 0.001 and = 0.0744, respectively), as shown in Table 2.

**Table 2:** The main clinical characteristics of patients with a ΔeGFR greater or less than 20% during the first year

	ΔeGFR < 20%	ΔeGFR ≥ 20%	p
Clinical characteristics			
Gender, female (%)	31.2	20.7	0.07
Baseline weight [kg]	127 ± 27	126 ± 27	0.73
Baseline BMI [kg/m^2^]	46.8 ± 9.3	47.4 ± 9.6	0.65
Baseline BSA [m^2^]	2.27 ± 0.26	2.24 ± 0.24	0.44
Hypertension, (%)	65.2	57.7	0.25
SBP [mmHg]	146 ± 24	145 ± 27	0.68
Diabetes, (%)	44.6	44.1	0.94
Laboratory results			
Glycemia [mg/dL]	103 ± 37	108 ± 40	0.32
Total cholesterol [mg/dL]	214 ± 48	208 ± 42	0.36
HDL- cholesterol [mg/dL]	44 ± 19	44 ± 15	0.95
LDL- cholesterol [mg/dL]	141 ± 48	137 ± 39	0.56
Triglycerides [mg/dL]	146 ± 68	152 ± 94	0.63
Hemoglobin [mg/dL]	14.6 ± 6.7	13.9 ± 6.1	0.42
Platelets [× 10^9^/L]	265 ± 73	257 ± 69	0.43
Immunoglobulins [g/L]	1.2 ± 0.3	1.3 ± 0.8	0.14
Albumin [g/dL]	4.1 ± 0.5	4.1 ± 0.5	0.86
Proteins [g/dL]	7.2 ± 0.6	7.3 ± 0.6	0.25
Kidney function			
Baseline creatinine [g/dL]	0.81 ± 0.25	0.93 ± 0.17	< 0.001
Baseline eGFR [ml/min/m^2^]	105 ± 18	90 ± 19	< 0.001
eGFR 1 year after BPD [ml/min/m^2^]	84 ± 16	99 ± 17	< 0.001
ΔGFR CKD-EPI unindexed. [ml/min/m^2^/y]	1.0 ± 6.8	8.2 ± 17.6	< 0.001
ΔBMI one year after BPD [%]	−30.0 ± 7.4	−33.6 ± 7.5	0.008
Annual ΔBMI [%/y]	−3.2 ± 4.4	−4.4 ± 5.9	0.07

Abbrevations: Δ, delta; ΔeGFR 1y, delta estimated glomerular filtration rate (calculated by unindexed CKD-EPI) at the 1 year time-point; BMI, body mass index; BSA, body surface area; SBP, systolic blood pressure

**Fig. 2:**
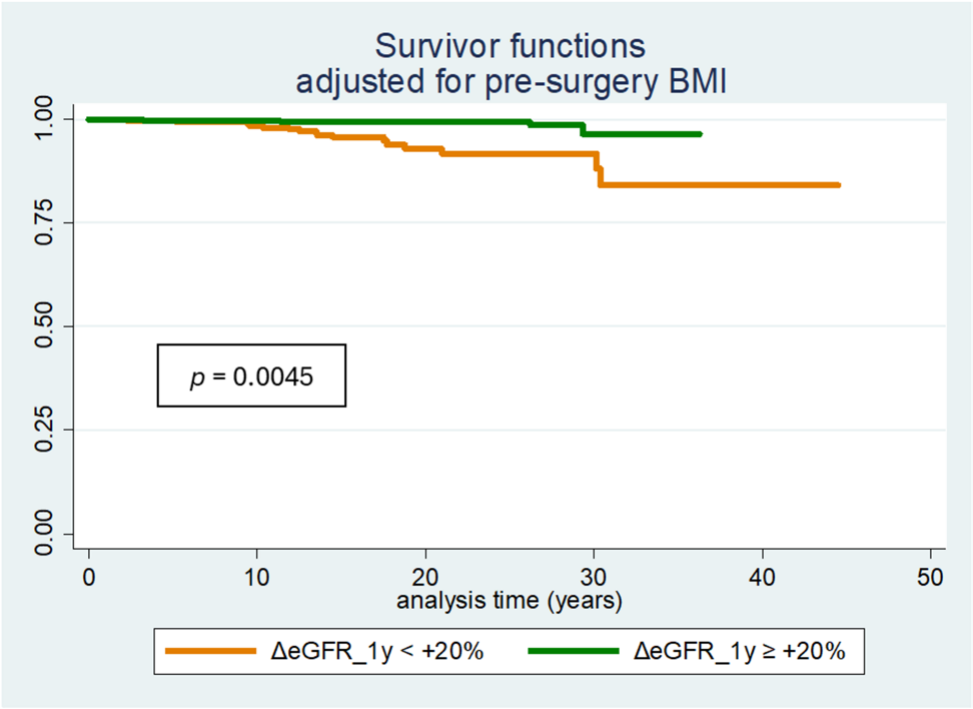
Kaplan–Meier survival curves on the basis of eGFR improvement during the first year. The analysis is adjusted for pre-surgery BMI. Abbreviations: deltaGFR, delta glomerular filtration rate (CKD EPI unindexed); BMI, body mass index

Patients who experienced an increase in eGFR greater than 20% during the first year after BPD had a better outcome, independently of basal BMI (Log Rank test *p* = 0.0045), as shown in Fig. 2.

Considering the entire follow-up period, the mean BMI showed an annual change of −3.7 ± 5% per year, while the unadjusted eGFR demonstrated an annual increase of + 3.1 ± 7.8 mL/min/m^2^. Patients with a lower ΔeGFR had lower preoperative weight, BMI, and BSA. Comorbidities did not significantly affect kidney function variation (Supplemental Table 2)*.*

**Fig. 3:**
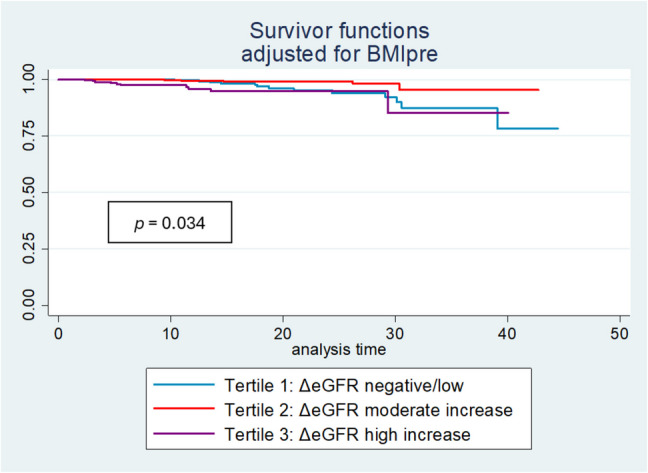
Kaplan–Meier survival curves on the basis of long-term eGFR changes. eGFR calculated by unindexed CKD-EPI. The analysis is adjusted for pre-surgery BMI. Abbreviations: deltaGFR, delta glomerular filtration rate (CKD EPI indexed); BMI, body mass index

Kaplan–Meier survival curves based on ΔeGFR tertiles (indexed CKD-EPI) and adjusted for pre-surgery BMI show that patients in the second tertile (ΔeGFR between + 0.5 and + 2.2 ml/min/year) have a significantly higher survival rate (log-rank *p* = 0.034) compared to those in the other tertiles (Fig. 3).

Both univariate and multivariate analyses indicate that baseline eGFR, BMI, and serum albumin do not have a significant impact on mortality. However,an elevation in eGFR over the very long term appears to be associated with significantly increased mortality (Table 3).

**Table 3:** Cox univariate and multivariate proportional hazard model for all-causes mortality

	Univariate			Multivariate		
	HR	95%CI	p	HR	95%CI	p
Age [years]	1.03	0.99–1.06	0.093	1.02	0.98–1.07	0.228
Female gender	1.31	0.60–2.89	0.495	1.14	0.38–3.39	0.813
eGFR at baseline [mL/min/m^2^]	0.99	0.98–1.02	0.889	0.99	0.96–1.04	0.948
BMI at baseline [kg/m^2^]	1.02	0.99–1.06	0.152	1.04	0.99–1.08	0.110
Serum Albumin [g/dL]	0.73	0.35–1.52	0.398	0.75	0.32–1.76	0.505
yearly increase of eGFR [mL/min/m^2^/y]	1.19	1.09–1.30	** < 0.001**	1.31	1.15–1.48	** < 0.001**
Increase of eGFR during the 1 st year [mL/min/m^2^]	0.98	0.97–1.00	0.059	0.96	0.93–0.98	**0.002**
Yearly decrease of BMI [Kg/m^2^/y]	3.18	1.70–5.98	** < 0.001**	2.48	1.01–6.07	**0.047**

Abbrevations: GFR, glomerular filtration rate (CKD EPI unadjusted, [ml/min/1.73m2]); BMI, body mass index pre-surgery (kg/m2); ΔeGFR yearly, yearly change in GFR after the first year; ΔBMI yearly, yearly change in body mass index after the first year

Indeed, the improvement in GFR during the first year after BPD appears to serve as a protective factor against mortality (HR 0.96 [95%CI 0.93–0.98], *p* = 0.002). In contrast, a higher annual ΔGFR beyond the first year of follow-up is associated with an increased risk of mortality (HR 1.31 [95%CI 1.15–1.48], *p* < 0.001). Furthermore, the annual decrease in BMI following surgery is linked to an independent increase in mortality risk (HR 2.48 [95%CI 1.01–6.07], *p* = 0.047).

## Discussion

This retrospective observational study highlights the intricate and time-dependent relationship between changes in eGFR and mortality risk following BPD. Notably, the timing of eGFR assessment emerged as a critical factor in understanding its prognostic implications. The lack of an early increase in eGFR and a long-term increase in eGFR are two unfavorable risk factors. While the former may indicate suboptimal outcomes of bariatric surgery, the latter may indicate malnutrition resulting from excessive muscle mass loss.

For people with very high BMI, BPD with duodenal switch resulted in greater weight loss than other procedures, resulting in greater improvement in weight loss outcomes and weight-associated comorbidities compared with non-surgical interventions [23, 24]. While sustained weight reduction after bariatric surgery leads to improvement in blood pressure and glycemic control, resulting in reduction in cardiovascular risk [25], the mean permanent reduction of about 75% of the initial excess weight following BPD is frequently associated with late complications including anemia, bone demineralization, and protein malnutrition [26]. Moreover, BPD is known to be associated with a decrease in serum creatinine and an early increase in eGFR, the prognostic effects in the long term remain to be investigated [27, 28].

Accurately evaluating GFR in patients with severe obesity, particularly following bariatric surgery, presents several challenges. First, commonly used equations for estimating GFR, such as the CKD-EPI and MDRD equations, were developed using older datasets from populations where severe obesity was far less prevalent. Second, both obesity and cachexia independently influence creatinine, regardless of GFR. Creatinine levels are directly linked to muscle mass, which decreases by 20–25% after BPD, opening the possibility for the development of malnutrition. Despite the advent of new specific formulas (i.e. EKCF, Salazar Corcoran) and attempts at indexing for real BSA [29, 30], the determination of GFR in these patients remains inconsistent, and no gold standard has been established yet. In this study, we found significant differences between GFR values calculated using different formulas and standard indexing compared to indexing with actual BSA. However, the trends remain similar in both the short- and long-term follow-up, providing consistency to the results (Fig. 1).

As previously reported in the literature, we found that the better GFR, the lower the prevalence of comorbidities, such as hypertension, diabetes, and more severe obesity (Table 1). In our cohort, renal hyperfiltration indicated by pathologically elevated eGFR values seems to be underrepresented, suggesting that severe obesity has already had time to cause long-term glomerular damage.

One of the key findings of our study is the dual impact of GFR changes on mortality risk.

We found that the lack of improvement in GFR (< + 20% compared to baseline) during the first year is a red flag for mortality risk in patients who underwent BPD (Fig. 2, Log Rank test *p* = 0.003). Several mechanisms, including weight loss, could account for the positive impact of surgery on renal function. While pre-surgical BMI was comparable between patients who experience improved kidney function and those who do not in the first year post-surgery, it was observed that patients with more than a 20% improvement in GFR lost a greater amount of body mass (−34% vs 30% of BMI in the first year, data not shown). Therefore, we can hypothesize that, in an early phase, greater body mass loss translates into benefits that include improved kidney function, consistent with previous studies [27, 31].

Interestingly, the results regarding eGFR changes during long-term diverged dramatically. Dividing the cohort into tertiles based on the annual change in eGFR, a greater increase in eGFR (> 2.2 mL/min/m^2^/year, 3° tertile) in the long term translates into an increased risk of mortality (Fig. 3). It is known that BPD requires a close follow-up and multiple vitamin and mineral supplements, especially regarding fat-soluble vitamins [32]. In some cases, nutritional consequences that may occur in bariatric patients can potentially undermine the clinical benefits of BPD [33]. In this context, an increase in GFR may not accurately represent true renal function; instead, it may be a result of decreased serum creatinine levels, which are directly associated with progressive muscle mass loss due to protein malnutrition. This paradoxical finding underscores the necessity of distinguishing true renal function improvement from potential artifacts by malnutrition-induced creatinine reductions. Our study has several limitations, alongside strengths that merit consideration. First, the retrospective observational design precludes causal inference, and only associations can be established. Second, the absence of directly measured GFR or alternative biomarkers such as cystatin C limits the precision of renal function assessment. Nevertheless, creatinine-based eGFR remains the most widely available and routinely used method in clinical practice, ensuring that our findings reflect real-world patient evaluation and follow-up, thereby maintaining high practical relevance.

Third, we acknowledge that changes in patient characteristics over time—including body weight, nutritional status, comorbidities, and medication use—may act as dynamic confounders affecting renal outcomes and survival. Due to the historical and retrospective nature of the cohort, with irregular follow-up intervals spanning several decades, fully addressing these time-varying confounders through advanced statistical modeling (e.g., marginal structural models or joint longitudinal-survival models) was not feasible. Consequently, our study did not implement a formal time-varying analysis, and proper adjustment for dynamic confounders was limited. Our analyses therefore relied primarily on baseline covariates and pre-specified eGFR thresholds, which may not capture the full impact of longitudinal changes.

Finally, the monocentric nature and relatively homogeneous population of the study limit generalizability to broader or more diverse patient groups.

Despite these limitations, the relatively large sample size, the exceptionally long follow-up period, and the inclusion of unindexed GFR calculated with the CKD-EPI formula make this study a unique and valuable contribution, potentially representing one of the first explorations of the long-term relationship between bariatric surgery and kidney function. Furthermore, BMI-adjusted data were analyzed to assess mortality risk in Kaplan-Meier analyses. A number of potentially confounding variables were considered, including age, gender, BMI, and albumin levels, strengthening the independent association between GFR changes and mortality. Therefore, the findings described herein provide critical insights into the nuanced relationship between bariatric surgery, renal function, and long-term mortality, paving the way for future research and improvement in clinical management.

Changes in eGFR have been identified as an independent predictor of mortality, with opposing effects depending on the timing of the measurement after surgery (Table 3).

In the short-term follow-up, GFR improvement within 1 year post BPD was associated with a reduced risk of mortality. However, in the long-term follow-up, for each annual ml/min improvement in eGFR we observed a 30% increased risk of mortality (Table 3).

The advent of GLP-1 receptor agonists (GLP-1 RAs) raises new questions about the future of bariatric surgery. These medications offer promising alternatives for sustained weight loss, potentially altering the risk-benefit profile of procedures like BPD. Moving forward, careful patient selection and close monitoring of risk factors—such as excessive weight loss or the absence of early eGFR improvement—will be essential in optimizing outcomes.

## Conclusions

This study demonstrates that changes in eGFR following BPD have distinct prognostic implications depending on the timing of assessment. The lack of early eGFR improvement predicts higher mortality risk, while long-term eGFR increases may reflect malnutrition and muscle mass loss. These findings highlight the importance of personalized postoperative monitoring and nutritional management to optimize patient outcomes. Given the advent of new weight-loss therapies, future research should compare their long-term renal effects with surgical interventions. Accurate GFR assessment methods tailored to severe obesity and bariatric surgery remain a priority for advancing clinical care.

## Supplementary Information

Below is the link to the electronic supplementary material.

ESM 1 (DOCX 23.1 KB)

## Data Availability

The data that support the findings of this study are available on request from the corresponding author. The data are not publicly available due to privacy or ethical restrictions.
