# Supplementary material for: Impact of GFR on Mortality Risk After Biliopancreatic Diversion: Challenges and Pitfalls for the Clinician
Source: Obes Surg. 2025 Sep 18;35(10):4362–9. doi: 10.1007/s11695-025-08239-z (PMC12540522; doi:10.1007/s11695-025-08239-z)
Supplement: Supplementary file 1 — (DOCX 23.1 KB) [file 11695_2025_8239_MOESM1_ESM.docx]

**SUPPLEMENTAL MATERIAL**

**Supplemental Table 1. different equations available for estimating glomerular filtration rate (GFR)**

| **Name** | **Gender** | **Age, years** | **Serum Creatinine** | **Equation** |
| --- | --- | --- | --- | --- |
| CKD-EPI | Female |  | ≤0.7 | 144×(SCr/0.7)^-0.329^×0.993^Age^ |
|  |  |  | >0.7 | 144×(SCr/0.7)^-1.209^×0.993^Age^ |
|  | Male |  | ≤0.9 | 141×(SCr/0.7)^-0.411^×0.993^Age^ |
|  |  |  | >0.9 | 141×(SCr/0.7)^-1.209^×0.993^Age^ |
| EKFC | Female | 18-40 | SCr<Q | 107.3×(SCr/Q)^-0.322^ |
|  |  |  | SCr≥Q | 107.3×(SCr/Q)^-1.132^ |
|  | Male | >40 | SCr<Q | 107.3×(SCr/Q)^-0.322^×0.990^(Age-40)^ |
|  |  |  | SCr≥Q | 107.3×(SCr/Q)^-1.132^×0.990 ^(Age-40)^ |
| COCKROFT-GAULT | Female |  |  | {[(140-Age) ×Wt]/SCr×72} ×0.85 |
|  | Male |  |  | [(140-Age) ×Wt]/SCr×72 |
| COCKROFT-GAULT  LEAN BODYWEIGHT | Female |  |  | {[(140-Age) ×Lean Wt]/SCr×72}  ×0.85 |
|  | Male |  |  | [(140-Age) ×Lean Wt]/SCr×72 |
| SALAZAR-CORCORAN | Female |  |  | {(137-Age)[(0.285×Wt)+(12.1×Ht^2^)]} / 51×SCr |
|  | Male |  |  | {(137-Age)[(0.285×Wt)+(12.1×Ht^2^)]} / 60×SCr |

Abbreviations: Wt=weight (kg); Ht=Height (m); SCr=serum creatinine (mg/dl); Q-values [=median serum creatinine] for the EKFC equation, according to age or height.

**Supplemental Table 2. Baseline** **clinical biochemical characteristics by annual eGFR tertiles.**

|  | All | ΔeGFR  1° tertile | ΔeGFR  2° tertile | ΔeGFR  3° tertile | p |
| --- | --- | --- | --- | --- | --- |
|  |  | < +0.5 ml/min/y | +0.5/+2.2 ml/min/y | > +2.2 ml/min/y |  |
| *General clinical characteristics* | | | | | |
| *Age [yrs]* | 36.1 ±10.9 | 37.3 ±11.7 | 34.2 ±9.8 | 36.5 ±10.9 | 0.161 |
| *Gender, F %* | 73.5 | 72.3 | 66.3 | 81.9 | 0.070 |
| *Weight pre [kg]* | 126 ±26 | 119 ±24 | 132 ±28 | 127 ±27 | 0.007 |
| *BMI pre [kg/m^2^]* | 47.0 ±9.2 | 44.5 ±8.1 | 48.3 ±8.9 | 48.1 ±10.0 | 0.099 |
| *BSA pre [m^2^]* | 2.25±0.25 | 2.20 ±0.23 | 2.31 ±0.27 | 2.25 ±0.24 | 0.015 |
| *Comorbidities* | | | | | |
| *Hypertension, %* | 60.2 | 61.4 | 61.4 | 57.8 | 0.86 |
| *SBP [mmHg]* | 146 ±27 | 149 ±25 | 146 ±26 | 143 ±29 | 0.377 |
| *Diabetes, %* | 43.0 | 44.5 | 45.8 | 38.5 | 0.602 |
| *Laboratory results* | | | | | |
| *Glycemia pre [mg/dL]* | 104 ±37 | 103 ±30 | 107 ±43 | 104 ±39 | 0.802 |
| *Cholesterol tot [mg/dL]* | 211 ±45 | 211 ±48 | 215 ±44 | 207 ±43 | 0.534 |
| *HDL- Cholesterol [mg/dL]* | 44 ±17 | 44 ±23 | 43 ±14 | 44 ±14 | 0.972 |
| *LDL- Cholesterol [mg/dL]* | 139 ±44 | 133 ±53 | 141 ±43 | 141 ±40 | 0.699 |
| *Triglycerides [mg/dL]* | 148 ±82 | 150 ±102 | 156 ±75 | 139 ±63 | 0.446 |
| *Hemoglobin [mg/dL]* | 14.2 ±6.1 | 13.8 ±1.6 | 13.8 ±1.5 | 14.9 ±10.3 | 0.439 |
| *Platelets [×10^9^/L]* | 262 ±70 | 265 ±74 | 254 ±67 | 268 ±70 | 0.401 |
| *Immunoglobulins [g/L]* | 1.28±0.56 | 1.24 ±0.31 | 1.16 ±0.22 | 1.43 ±0.86 | 0.014 |
| *Serum Albumin pre [g/dL]* | 4.1 ±0.5 | 4.0 ±0.5 | 4.1 ±0.6 | 4.2 ±0.5 | 0.199 |
| *Proteins tot [g/dL]* | 7.2 ±0.6 | 7.1 ±0.7 | 7.2 ±0.5 | 7.3 ±0.5 | 0.061 |
| *Renal function* | | | | | |
| *Creatinine pre [g/dL]* | 0.87±0.21 | 0.80 ±0.25 | 0.88 ±0.20 | 0.93 ±0.17 | <0.001 |
| *GFR CKD-EPI pre [ml/min/1.73m^2^]* | 75.5±15.9 | 82.8 ±16.8 | 75.3 ±13.7 | 68.4 ±13.9 | <0.001 |
| *Annual ΔBMI [%/y]* | -3.7 ±5.0 | -2.4 ±3.1 | -1.9 ±1.3 | -6.7 ±7.1 | <0.001 |
| *ΔGFR EPI unindexed [ml/min/1.73m^2^/y]* | 3.1 ±7.8 | -0.8 ±1.9 | 1.2 ±0.5 | 8.8 ±11.2 | <0.001 |

GFR estimated by unindexed CKD-EPI)

Abbrevations: ΔeGFR, yearly change in GFR over time; GFR, glomerular filtration rate (CKD EPI) BMI, body mass index; BSA, body surface area; SBP, sistolic blood pressure.
